# Sero-epidemiological status and risk factors of toxoplasmosis in pregnant women in Northern Vietnam

**DOI:** 10.1186/s12879-019-3885-7

**Published:** 2019-04-18

**Authors:** G. Suzanne A. Smit, Binh Thi Lam Vu, Dung Trung Do, Quan Ha Do, Huy Quang Pham, Niko Speybroeck, Brecht Devleesschauwer, Elizaveta Padalko, Ellen Roets, Pierre Dorny

**Affiliations:** 1 Department of Virology, Parasitology and Immunology, Faculty of Veterinary Medicine, Ghent University, Salisburylaan 133, 9820 Merelbeke, Belgium; 2 Department of Biomedical Sciences, Institute of Tropical Medicine (ITM), Nationalestraat 155, B-2000 Antwerp, Belgium; 3 Institute of Health and Society (IRSS), Université catholique de Louvain, Clos Chapelle-aux-champs 30, 1200 Woluwe-Saint Lambert, Brussels, Belgium; 4 Parasitology Department of the National Institute of Malariology, Parasitology and Entomology (NIMPE), 245 Luong The Vinh, Nam Tu Liem, Ha Noi Viet Nam; 5 National Hospital of Obstetrics and Gynaecology, 43 Trang Thi street, Hoan Kiem, Ha Noi Viet Nam; 6 Obstetrics and Gynaecology Hospital of Thai Binh, 530A Ly Bon street, Thai Binh city, Thai Binh province Viet Nam; 7 Department of Epidemiology and Public Health, Sciensano, Rue Juliette Wytsmanstraat 14, 1050 Brussels, Belgium; 8 Department of Veterinary Public Health and Food Safety, Faculty of Veterinary Medicine, Ghent University, Salisburylaan 133, 9820 Merelbeke, Belgium; 9 Department of Clinical Biology, Microbiology and Immunology, Ghent University Hospital, C. Heymanslaan 10, 9000 Ghent, Belgium; 10 School of Life Sciences, Hasselt University, Agoralaan Building D, 3590 Diepenbeek, Belgium; 11 Women’s Clinic, Ghent University Hospital, C. Heymanslaan 10, 9000 Ghent, Belgium

**Keywords:** Toxoplasmosis during pregnancy, Congenital toxoplasmosis, Seroprevalence, Risk factors, Prevention, Vietnam

## Abstract

**Background:**

In Vietnam, few studies have determined the epidemiological status of toxoplasmosis in pregnant women and no routine prenatal screening is in place. This study was conducted to evaluate the seroprevalence of this zoonotic parasitic infection in pregnant women in Northern Vietnam and to assess the association with awareness, risk factors and congenital toxoplasmosis.

**Methods:**

Approximately 800 pregnant women were included in the study from two hospitals, one in Hanoi and one in Thai Binh province, which is known to have a dense cat population. Serological immunoglobulin G (IgG) and immunoglobulin M (IgM) detection was performed to estimate the seroprevalence of toxoplasmosis and sero-incidence of maternal and congenital toxoplasmosis. In addition, a survey was conducted about awareness, clinical history, presentation of signs and symptoms relating to toxoplasmosis and to detect biologically plausible and socio-demographic risk factors associated with toxoplasmosis. Associations with seroprevalence were assessed using univariable and multivariable analysis.

**Results:**

The mean IgG seroprevalence after the full diagnostic process was 4.5% (95% confidence interval(CI): 2.7–7.0) and 5.8% (95% CI: 3.7–8.6) in Hanoi and Thai Binh hospital, respectively, and included one seroconversion diagnosed in Thai Binh hospital. Only 2.0% of the pregnant women in Hanoi hospital and 3.3% in Thai Binh hospital had heard about toxoplasmosis before this study.

**Conclusion:**

Since the percentage of seronegative, and thus susceptible, pregnant women was high and the awareness was low, we suggest to distribute information about toxoplasmosis and its prevention among women of child bearing age. Furthermore, future studies are recommended to investigate why such a low seroprevalence was seen in pregnant women in Northern Vietnam compared to other countries in South East Asia and globally.

**Electronic supplementary material:**

The online version of this article (10.1186/s12879-019-3885-7) contains supplementary material, which is available to authorized users.

## Background

*Toxoplasma gondii* is a zoonotic protozoan parasite with a worldwide distribution. Warm-blooded animals, including humans, are intermediate hosts and felids are the definitive hosts. Based on the *T. gondii* life cycle, it can be predicted that humans can acquire toxoplasmosis by infection with cat-shed oocysts due to different kinds of contacts with the environment contaminated by sporulated oocysts, including soil, food, and water or with tissue cysts by consuming raw or undercooked infected meat. The relative importance of different routes of transmission in different regions remains unclear [1, 2], yet variation is seen in the human toxoplasmosis seroprevalence between and within countries. A large part of the variability can be explained by factors such as alimentary habits; the presence of cats; hygienic conditions and habits; the climate; economic, social or cultural habits; and water quality [3–7].

Acquired toxoplasmosis is usually asymptomatic or results in a relatively mild acute illness in immunocompetent individuals, with some cases suffering from acquired chorioretinitis (also referred to as retinochoroiditis) and/or fatigue, yet it can cause serious disease in immunocompromised patients [8]. There is increasing evidence that chronic toxoplasmosis may also result in a number of psychiatric or neurological diseases even in immunocompetent individuals [9, 10].

Congenital toxoplasmosis (CT) is caused by transplacental transmission of tachyzoites to the unborn child. Women who are seropositive have negligible risk for CT because it mainly occurs when a woman is primarily infected with *T. gondii* during pregnancy. Congenital toxoplasmosis is asymptomatic in most cases but it can also result in congenital defects, such as hydrocephalus, central nervous system abnormalities, chorioretinitis and even fetal or neonatal death [11–13]. The estimated global incidence of CT is 1.5 (95% confidence interval (CI): 1.4–1.6) cases per 1000 live births, which resulted in an estimated public health impact of 9.6 (95% CI: 5.8–15) Disability-Adjusted Life Years (DALYs) per 1000 live births [13]. Since simple primary prevention measures can reduce the risk of infection during pregnancy in seronegative women, it can be important to know her serological status at the beginning of pregnancy [14]. For these measures to be successful in the local context it is important to know the major risk factors associated with the infection.

Only few studies have determined the epidemiological status of toxoplasmosis in pregnant women and no studies have assessed the risk factors associated with toxoplasmosis in Vietnam, a densely populated country in southeast Asia with a population of around 93.5 million in 2015 [15]. As far as we know, no systematic prenatal screening nor prevention measures for toxoplasmosis are in place and the awareness is low in this country. Therefore, this study aimed to evaluate the seroprevalence of toxoplasmosis in pregnant women in hospitals in Hanoi and Thai Binh, Northern Vietnam, and to assess the association with awareness, risk factors and CT.

## Methods

### Study design and setting

The two study sites were the National Hospital of Obstetrics and Gynaecology in Vietnam’s capital Hanoi, one of the leading hospitals in Vietnam for obstetrics and gynaecology, and the Hospital of Obstetrics and Gynaecology in Thai Binh province (120 km south east of Hanoi), in the remainder of this paper referred to as Hanoi hospital and Thai Binh hospital, respectively. Both hospitals are public hospitals, accessible for women of all layers of the society. Hanoi and Thai Binh province have an approximate population size of 7.2 million and 1.8 million, respectively [15]. Thai Binh is known as the “cat province” because of its dense cat population - pets, stray cats, and cats for meat consumption - in contrast to the rest of Vietnam [16]. More background information, eligibility criteria and the full study protocol are described in Smit et al. [17].

From October 2016 to March 2017 the gynaecologists from both hospitals identified approximately 800 eligible pregnant women attending antenatal care for the first time within their current pregnancy. At first consult, participants were asked to fill in a structured questionnaire concerning clinical history, awareness, presentation of signs and symptoms related to toxoplasmosis, and potential socio-demographic and biologically plausible risk factors (Additional files 1 and 2). Awareness was raised on the importance of toxoplasmosis and its possible consequences on pregnancy and a leaflet (in Vietnamese) was handed out explaining (congenital) toxoplasmosis and its prevention for further read-up and reminder at home. In addition, 5 ml blood were collected from participating women by the medical technicians in the hospital for further serological analysis.

### Laboratory procedures

All serum samples were tested for specific anti-*Toxoplasma* immunoglobulin G (IgG) by the Toxoscreen Direct Agglutination kit (BioMérieux, Marcy-l’Etoile, France; relative sensitivity (compared to Dye-Test) 96.22% (95% CI 94.55–97.39) and relative specificity 98.80% (95% CI 96.46–99.60) according to manufacturer’s instructions) on 1/40 and 1/4000 serum dilutions. In addition, an IgM test was conducted using the ISAGA kit (Immunoglobulin-M immunosorbent agglutination assay, BioMérieux, Marcy-l’Etoile, France) to detect recent infection. Detection of IgM and IgG is possible from approximately 2 and 4 weeks after infection, respectively. IgG persists lifelong in immunocompetent people whereas IgM is detectable by ISAGA for approximately a year (median 12.8 months, interquartile range 6.9–24.9) [18–20].

Women who tested positive for IgG only were considered seropositive. Women who were IgM positive were tested again for IgG and IgM 3–4 weeks later. When positive for IgG and IgM in the first test an IgG avidity test (Roche Diagnostics; on the first serum sample; performed in the National Hospital of Obstetrics and Gynaecology in Hanoi) was done to determine the time of seroconversion. An IgG negative and IgM positive test result in the first test and an IgG positive test result 3–4 or 6–8 weeks later was considered indicative for seroconversion. At any suspicion of seroconversion, women were advised and followed-up (including an ultrasound every 4 weeks) by their treating gynaecologist.

Children were thoroughly investigated and followed-up for any signs of CT by a neonatologist/paediatrician of the parents’ choice when there was an indication of seroconversion during pregnancy. For serology, blood samples were collected and tested for toxoplasmosis specific IgG, IgM, and IgA (performed in Belgium; Platelia™ Toxo IgA, Bio-Rad). The presence of IgM and IgA in neonatal serum is diagnostic for CT but the sensitivity is low and decreases when the infection occurred early during pregnancy [21, 22]. Persisting IgG antibodies 9 months to 1 year after birth are an indication for CT but only if the mother is also found IgG positive in the perinatal period [21]. More information about the laboratory procedures and the full study protocol including (diagnostic) follow-up are described in Smit et al. [17].

### Statistical analysis

Data from the source documents were entered in Microsoft Excel 2011 (Microsoft, Redmond, United States of America). The populations were divided in seronegative (susceptible) and seropositive (infected, i.e. seroconversion, or recovered, i.e. IgG positive only) individuals, the latter with (at least IgG) humoral immunity exceeding the threshold of the fixed diagnostic cut-off values provided by the manufacturers of the assays, implying (past) infection. Hence, the immunological status of the individual follows a Bernoulli distribution and the mean seroprevalence of toxoplasmosis in pregnant women in Hanoi and Thai Binh hospital could be determined.

The questionnaire was analysed to detect biologically plausible and socio-demographic risk factors associated with toxoplasmosis, clinical history, awareness and presentation of signs relating to toxoplasmosis. Association between the seroprevalence of *T. gondii* infection and possible demographic and risk factors were explored using univariable and multivariable analysis. A generalized linear model with a binomial distribution and logit link function was applied. For categorical variables, the Pearson’s chi-square test was used as a goodness of fit to proof that the observed data did not differ from the theoretical distribution. When any cell value had < 5 observations and/or a separation problem occurred for a variable, a logistic regression model using Firth’s bias reduction method and the Fisher’s exact test were used [23]. Variables with a *P* value under the threshold *P* ≤ 0.20 were analysed in a multivariable model. The multivariable model was constructed using a logistic regression model using Firth’s bias reduction method, backwards selection and based on a significance level for inclusion of *P* ≤ 0.05.

The Clopper-Pearson method was used to estimate the binomial confidence interval and as such to summarize the statistical uncertainty about the seroprevalence by the mean and 95% CI. All calculations were performed in R 3.5.0 (R Core Team 2018) [24].

## Results

In total, 402 eligible pregnant women were recruited in Hanoi hospital and 397 in Thai Binh hospital. Every participant was informed on prevention measures, the diagnostic test results and was offered appropriate medical information and medical follow-up if required. We found 17 women in Hanoi and 21 in Thai Binh hospital seropositive for toxoplasmosis IgG only at first visit. In Hanoi hospital four women were followed-up based on a positive IgM and negative IgG result (*n* = 3) or both IgM and IgG positive result (*n* = 1). In Thai Binh hospital seven women had a positive IgM result only (*n* = 6) or both IgM and IgG positive result (*n* = 1). Three women started treatment with spiramycine within the medical follow-up, of which one woman continued this treatment until delivery based on suspicion of a seroconversion. The two samples that showed IgG and IgM positivity showed a high avidity, which suggested an old infection, and were subsequently considered seropositive. Within the follow-up a false positive IgM result was concluded for two samples in Hanoi and two samples in Thai Binh, and were subsequently considered seronegative. In these IgM false positive cases, the gestational age at the first blood sample ranged between 9 and 13 weeks. After the first tests with IgG negative and IgM positive results, a second blood sample was taken 4–5 weeks later, which tested IgG negative and IgM negative. In all but one case, these IgM negative results were confirmed by an IgM ELISA at the National Hospital of Obstetrics and Gynaecology Hanoi.

Four women did not want to be followed-up and dropped out before a final conclusion could be made on their serological status. However, we tried to remain in contact and provided information and diagnostic testing when requested. All pregnancies within the follow-up were without abnormalities and the newborns were considered healthy. One newborn, from the mother who had been under treatment during pregnancy, was at the time of writing followed-up based on suspicion of congenital infection. The serum samples from the first 3 months of this newborn were both IgM and IgA negative and showed a decreasing IgG titer, which might mean that no congenital infection had taken place. However, the final diagnosis can only be made at 1 year after birth. The patient received proper consultation and medical follow-up by a pediatrician.

Taking into account all diagnostic results of the women who remained in the study (*n* = 401 in Hanoi hospital and *n* = 394 in Thai Binh hospital), the mean seroprevalence was 4.5% (95% CI: 2.7–7.0) and 5.8% (95% CI: 3.7–8.6) in Hanoi and Thai Binh hospital, respectively. The mean age of these women was 27 years (standard deviation (sd): 5) in Hanoi and 28 years (sd: 5) in Thai Binh hospital.

Information regarding seroprevalence, age, clinical history, presentation of signs and symptoms, awareness and the presence of cats from the questionnaire is summarized in Table 1. The questionnaire was analysed to detect socio-demographic and biologically plausible risk factors associated with toxoplasmosis. The complete results of the univariable analyses are presented in Additional file 3, while the significant associations of the univariable and multivariable models are summarized in Table 2 and Table 3 for Hanoi and Thai Binh hospital, respectively.

**Table 1:** Descriptive statistics of toxoplasmosis and other variables in pregnant women in Hanoi and Thai Binh hospital

Variable	Hanoi hospital	Thai Binh hospital
Mean seroprevalence	4.5% (95% CI: 2.7–7.0)	5.8% (95% CI: 3.7–8.6)
Mean age	27 years (sd: 5)	28 years (sd: 5)
Mean gestational weeks	12 weeks (sd: 1)	10 weeks (sd: 3)
Mean previous pregnancies	1.2 (sd: 1.1)	1.3 (sd: 1.1)
Previous stillbirths	32% (127/401)	36% (141/394)
Mean number of previous stillbirths	1.4 (sd: 0.6)	1.3 (sd: 0.6)
Symptoms potentially relating to toxoplasmosis	6.5% (26/401)	5.6% (22/394)
Heard about toxoplasmosis before	2.0% (8/401)	3.3% (13/394)
*From a doctor*	1.0% (4/401)	2.0% (8/394)
*From the internet*	0.5% (2/401)	1.8% (7/394)
*From TV*	0.5% (2/401)	No
*From studying medicine*	0.3% (1/401)	No
Owning a cat	28% (112/401)	39% (152/394)
(Stray) cats on the property/neighbourhood/ work environment	30% (122/401)	35% (138/393)

Abbreviations: *CI* confidence interval, *sd* standard deviation

**Table 2:** Univariable and multivariable analysis of possible demographic and risk factors for toxoplasmosis in Hanoi hospital

Variable	IgG seronegative No. (%) or mean (sd)	IgG seropositive No. (%) or mean (sd)	Odds Ratio (95% CI)	*P*-value
Univariable analysis				
Gestational weeks	12 (1)	13 (1)	2.49 (1.20–5.18)	0.015
Government employed				0.130
*No*	229 (97.0%)	7 (3.0%)	–	
*Yes*	154 (93.3%)	11 (6.7%)	2.34 (0.886–6.16)	
Consumption of chicken or duck				0.046^a,b^
*No*	28 (87.5%)	4 (12.5%)	–	
*Yes*	355 (96.2%)	14 (3.8%)	0.258 (0.083–0.806)	
Consumption of cat meat				0.091
*No*	324 (96.4%)	12 (3.6%)	–	
*Yes*	59 (90.8%)	6 (9.2%)	2.75 (0.992–7.60)	
Household tasks related contact with soil/sand/floor/pavement/ street				0.0733
*No*	311 (96.6%)	11 (3.4%)	–	
*Yes*	72 (91.1%)	7 (8.9%)	2.75 (1.03–7.34)	
Multivariable analysis				
Gestational weeks	12 (1)	13 (1)	2.29 (1.15–4.59)	0.019^b^
Government employed	154 (93.3%)	11 (6.7%)	3.11 (1.14–8.49)	0.027^b^
Consumption of chicken or duck	355 (96.2%)	14 (3.8%)	0.191 (0.056–0.648)	0.008^b^
Household tasks related contact with soil/sand/floor/pavement/ street	72 (91.1%)	7 (8.9%)	2.65 (1.00–7.01)	0.050^b^

^a^Fisher’s exact test; ^b^Obtained using Firth’s bias reduction method [20]

Abbreviations: *CI* confidence interval, *sd* standard deviation

**Table 3:** Univariable and multivariable analysis of possible demographic and risk factors for toxoplasmosis in Thai Binh hospital

Variable	IgG seronegative No. (%) or mean (sd)	IgG seropositive^#^ No. (%) or mean (sd)	Odds Ratio (95% CI)	*P*-value
Univariable analysis				
Level of education				0.157^a,b^
*College*	92 (93%)	7 (7%)	–	
*Preschool*	1 (100%)	0 (0%)	4.11 (0.042–404)	
*Primary school*	9 (75%)	3 (25%)	4.54 (1.04–19.9)	
*Secondary school*	94 (97%)	3 (3%)	0.457 (0.124–1.69)	
*High school*	85 (93%)	6 (7%)	0.938 (0.313–2.81)	
*University degree*	87 (96%)	4 (4%)	0.634 (0.189–2.13)	
*Post university degree*	3 (100%)	0 (0%)	1.76 (0.053–58.5)	
Street cleaning profession				0.114^a,b^
*No*	370 (94.4%)	22 (5.6%)	–	
*Yes*	1 (50.0%)	1 (50.0%)	16.5 (0.997–272)	
Consumption of pork				0.0095^a,b^
*No*	1 (33.3%)	2 (66.7%)	–	
*Yes*	370 (94.6%)	21 (5.4%)	0.035 (0.003–0.375)	
Consumption of chicken or duck				0.114^a,b^
*No*	1 (50.0%)	1 (50.0%)	–	
*Yes*	370 (94.4%)	22 (5.6%)	0.061 (0.004–1.00)	
Frequency meat consumption per week	10 (3)	9 (4)	0.906 (0.803–1.02)	0.108
River as usual source of water				0.058^a,b^
*No*	371 (94%)	22 (6%)	–	
*Yes*	0 (0%)	1 (100%)	49.5 (0.525–4670)	
Multivariable analysis				
Street cleaning profession	1 (50.0%)	1 (50.0%)	18.0 (1.09–299)	0.044^b^
Consumption of pork	370 (94.6%)	21 (5.4%)	0.033 (0.003–0.359)	0.005^b^

^#^Including seroconversion; ^a^Fisher’s exact test; ^b^Obtained using Firth’s bias reduction method [20]

Abbreviations: *CI* confidence interval, *sd* standard deviation

The data showed that, with every increase in gestational weeks, women in Hanoi hospital had 2.29 (95% CI: 1.15–4.59) higher odds to test seropositive. Being employed by the government showed 3.11 (95% CI: 1.14–8.49) higher odds, household tasks related contact with soil, sand, floor, pavement or street was associated with 2.65 (95% CI: 1.00–7.01) higher odds, and a negative association with toxoplasmosis seroprevalence was observed when chicken or duck was consumed (0.191 (95% CI: 0.056–0.648)) . In Thai Binh hospital, pregnant women with the profession “street cleaning” showed 18.0 (95% CI: 1.09–299) higher odds to test seropositive and 0.033 (95% CI: 0.003–0.359) lower odds when they consumed pork. In both hospital populations we did not find an association between the seroprevalence and people owning a cat or having (stray) cats on the property/ neighbourhood/ work environment. Among cat owners there were no significant variables observed neither in the univariable nor in the multivariable model.

## Discussion

Since a noticeable impact of primary prevention on the burden of CT was observed by Smit et al. [25], we estimated the sero-epidemiological status and risk factors of toxoplasmosis in pregnant women in Northern Vietnam, a region with an assumed low level of awareness and lack of prevention measures. The mean estimated seroprevalence of 4.5% (95% CI: 2.7–7.0) and 5.8% (95% CI: 3.7–8.6) in Hanoi and Thai Binh hospital, respectively, were surprisingly low. With alimentary habits of eating raw/medium rare meat and raw vegetables and the presence of cats, we would expect the seroprevalence in pregnant women in Vietnam to be similar to for example the European seroprevalence, within the approximate range of 10–50% in pregnant women [26]. Studies conducted between 1959 and 2003 in Vietnam showed an overall low, yet higher toxoplasmosis seroprevalence compared to our study, with 11% in pregnant women, and ranging from 7.7 to 29% in the general population [27, 28]. Yet, a similar seroprevalence of 4.2% was found in a sero-survey in 2006 on toxoplasmosis in rural areas of the northern provinces, Nghe An, Lao Cai and Tien Giang [29]. In animals, the seroprevalence has been studied in pigs (27%) [30] and in cattle and water buffaloes (11 and 3.0%, respectively) [31] but, to our knowledge, not in other animals, such as cats. Even though large variability within and between countries in South East Asia was reported before, the seroprevalence found in the current study was low compared to other countries in this region and globally [26], especially considering the alimentary habits and presence of cats. Using standard commercial ELISA kits a seroprevalence of 43% (95% CI: 36–49) was observed in Malaysian pregnant women, 31% (95% CI: 28–37) in Myanmarese pregnant women [32] and 25% (95% CI: 22–28) in pregnant women in Southern Thailand [33]. Examples of studies that showed similar low seroprevalence in pregnant women in this region were conducted in Thailand, Bangkok (ELISA: 5.3% (95% CI: 3.8–6.8)) [34] and China, Nanning, Guangxi (indirect hemagglutination test: 7.0%). In China, one of the lowest seroprevalence estimates worldwide were reported, even below 1% in some south-western provinces [35].

The low seroprevalence means that the majority of pregnant women in Northern Vietnam were seronegative, and thus susceptible, which might make dissemination of information about primary prevention important, especially since very few pregnant women have heard about toxoplasmosis and how it can be acquired (only 2% in Hanoi hospital and 3.3% in Thai Binh hospital). However, low seroprevalence might also imply a low risk of infection for pregnant women. There may be a trade-off between seroprevalence, force of infection, and average age of pregnancy. To accurately model this, a larger sample size would be required. Either way, since dissemination of information about toxoplasmosis and its prevention is relatively easy and cheap, we would suggest distributing this among women of childbearing age.

Although we found a (non-significantly) higher toxoplasmosis seroprevalence in Thai Binh hospital, we could not conclude that the seroprevalence in both survey sites was associated to people owning a cat or to having (stray) cats on the property/ in the neighbourhood/ work environment. Pappas et al. [26] and Petersen et al. [1] already noticed that a surprisingly absent risk factor in most studies was contact with cats. Direct contact with *T. gondii* shedding cats might not result in toxoplasmosis since oocysts passed in their faeces are unsporulated and, thus, not immediately infective. However, after sporulation in the environment they are a source of infection [1, 4].

A clear limitation was that the logistic regression necessarily needs sufficient limiting sample size (in our study the number of seropositives). Peduzzi et al. [36, 37] suggested that logistic models produce reasonably stable estimates if the limiting sample size has approximately 10 to 15 events per predictor. In the majority of the variables this was not met, so caution is needed for statistical inference. This may also explain the few and unexpected significant associations (e.g. negative associations with the consumption of chicken or duck in Hanoi and pork consumption in Thai Binh). In addition, the significantly associated binary variables in the multivariable models, and many of the binary variables analysed in the univariable models, had a small number of observations, or in contrast a very large number of observations, which made the variables unbalanced. For example, in Thai Binh there were 2/394 pregnant women with a street cleaning profession, of which one was seropositive, and 3/394 answered they never ate pork, of which two were seropositive. Finally we cannot fully rule out confounders.

Extrapolation of the results for pregnant women in Hanoi and Thai Binh and by extension for Northern Vietnam might induce selection bias. However, since these hospitals are accessible for women of all layers of the society and women in Vietnam, especially in urban areas, tend to go the gynaecologist from the moment they suspect to be pregnant and go for follow up consultation and ultrasound every month until delivery, these two hospital populations might be considered representative. In addition, it is unlikely that an overrepresentation of women with potential complications occurred in the study, since we only included pregnant women attending antenatal care for the first time within their current pregnancy.

Our study might have included some information bias due to the diagnostic test performances. In case of a seroconversion the ISAGA ensures very early detection of IgM yet the test is very sensitive to residual IgM, which can persist for more than 1 year (according to the manufacturer). By retesting and following up the patients, conducting confirmatory tests, complementing the results with additional diagnostic techniques, interpretation of the results taking into account the results of all (other) tests performed and the patient’s history, and thorough consultation and discussion with all stakeholders involved, our protocol [17] took this into account.

## Conclusion

The mean estimated toxoplasmosis seroprevalence in pregnant women in Hanoi and Thai Binh hospital was surprisingly low. Since the percentage of seronegative, and thus susceptible, pregnant women was high and the awareness was low, we suggest to increase awareness and distribute information about toxoplasmosis and its prevention among pregnant women at first consult and preferably even before pregnancy to reduce the prevalence and risk of transmission of this zoonosis. It would be interesting to investigate why such a low seroprevalence was seen in pregnant women in Northern Vietnam compared to other countries in South East Asia and globally. Further research could include investigation of the *T. gondii* prevalence in cats and livestock, investigation of the *T. gondii* strains involved, and the susceptibility of humans and/or warm-blooded animals in this region.

## Additional files

Additional file 1: Questionnaire-English. (PDF 136 kb)

Additional file 2: Questionnaire-Vietnamese. (PDF 587 kb)

Additional file 3: Univariable analysis. (PDF 277 kb)

## Abbreviations

CI: Confidence interval
DALY: Disability-Adjusted Life Year
IgG: Immunoglobulin G
ITM: Institute of Tropical Medicine
NIMPE: National Institute of Malariology, Parasitology and Entomology
SD: Standard deviation
