# Supplementary material for: Sero-epidemiological status and risk factors of toxoplasmosis in pregnant women in Northern Vietnam
Source: BMC Infect Dis. 2019 Apr 18;19:329. doi: 10.1186/s12879-019-3885-7 (PMC6471880; doi:10.1186/s12879-019-3885-7)
Supplement: Supplementary file 1 — Questionnaire-English. (PDF 136 kb) [file 12879_2019_3885_MOESM1_ESM.pdf]

Additional file 1

Questionnaire

Patient code:

Interviewer name: ____

Interviewer hospital: ____

Date: ________________

Section I: Introduction to the study

The interest of this research project is the parasite *Toxoplasma gondii.*
The aim of this questionnaire is to gain knowledge of the risk factors associated with toxoplasmosis, your clinical history and presentation of signs and symptoms relating to toxoplasmosis and awareness.
The study has been approved by the Ethical Committee of the Vietnamese Ministry of Health, by the Institutional Review Board of the Institute of Tropical Medicine and the Ethical Committee of the University Hospital in Antwerp, Antwerp, Belgium.

Section II: Interviewee ID

1. Age: ___________________
2. Place of residence/Zip code: ____
3. Level of education:
   - □ Preschool
   - □ Primary school
   - □ Secondary school
   - □ High school
   - □ College
   - □ University degree
   - □ Post university degree
4. Profession: (*Checking more than one checkbox is possible*)
   - □ Housewife
   - □ Farmer
   - □ Agricultural sector
   - □ Slaughter house worker
   - □ Butcher
   - □ Street cleaner

- ☐ Builder
- ☐ Government employed
- ☐ Business women
- ☐ Other Please Specify: ___________________________

Section III: Clinical history

5. Gestational weeks: ____
6. Number of previous pregnancies: ____________________________________
7. Previous stillbirths: No ☐ Yes ☐ please specify:___________________________
8. Did you have any of the following conditions during your current pregnancy? (*Checking more than one checkbox is possible*)
   - ☐ Mononucleosis like symptoms *(e.g. bilateral, non-tender cervical or axillary lymphadenopathy, flu-like syndrome like fever, malaise, myalgia, hepatosplenomegaly, and pharyngitis)*
   - ☐ Chorioretinitis *(e.g. ocular pain, blurred vision, blindness)*
   - ☐ Central nervous system abnormalities *(e.g. headache, altered mental status, seizures, coma, fever, focal neurologic deficits, such as motor or sensory loss, cranial nerve palsies, visual abnormalities, and focal seizures)*
   - ☐ None of the above
   - ☐ Other, please specify: ____

Section IV: Awareness

9. Have you heard about toxoplasmosis? Yes ☐ No ☐
10. Where did you obtain this knowledge? (*checking more than one checkbox is possible*)
    - ☐ Doctor
    - ☐ Internet
    - ☐ Peers
    - ☐ Other, please Specify: ___________________________

Section V: Eating/cooking habits

11. Which meat do you consume (specify how often)?

(*Checking more than one checkbox is possible*)

◻	Pork	☐ Never ☐Sometimes ☐ Often
◻	Beef	☐ Never ☐Sometimes ☐ Often
◻	Goat	☐ Never ☐Sometimes ☐ Often
◻	Chicken/Duck	☐ Never ☐Sometimes ☐ Often
◻	Fish	☐ Never ☐Sometimes ☐ Often
◻	Dog	☐ Never ☐Sometimes ☐ Often
◻	Cat	☐ Never ☐Sometimes ☐ Often
◻	Other, please specify: ______________________________	
		☐ Never ☐Sometimes ☐ Often

12. On average, how many times per week do you consume meat?

☐ 1 time ☐ 2 times ☐ 3 times ☐ 4 times ☐5 times ☐ 6 times ☐ 7 times ☐ More often: _____

13. How do you consume your meat? (*Checking more than one checkbox is possible*)

◻	Raw	☐ Never ☐Sometimes ☐ Often
◻	Medium rare	☐ Never ☐Sometimes ☐ Often
◻	Well done	☐ Never ☐Sometimes ☐ Often
◻	Cured/smoked/fermented	☐ Never ☐Sometimes ☐ Often
◻	Bbq	☐ Never ☐Sometimes ☐ Often
◻	Microwave	☐ Never ☐Sometimes ☐ Often

14. Do you freeze meat before consumption?

☐ Never ☐ Sometimes ☐ Often ☐ Always

15. Do you eat raw vegetables, fruits and/or salads?

☐ Never ☐ Sometimes ☐ Often

16. Do you wash vegetables, fruits and/or salads before you eat them raw?

☐ Never ☐ Sometimes ☐ Often ☐ Always

17. When do you wash your hands during the day?

(*Checking more than one checkbox is possible*)

◻ Before eating
◻ Before preparing food
◻ After gardening
◻ After cleaning the house/floor/pavement
◻ After cleaning the cat litter box/ cat faeces
◻ After handling something dirty
◻ After using the toilet

- ☐ Never
- ☐ Other, please specify: ______________________________

18. With what do you wash your hands?

- ☐ Water only
- ☐ Water with soap

19. How do you wash your hands?

- ☐ Water in a basin where hands are washed (*multiple people use the same water)*
- ☐ Water from a tap for pouring on hands (*single use)*
- ☐ Water in a container for pouring on hands (*single use)*
- ☐ Other, please specify: ______________________________

20. What is your usual source of water for daily use?

(*Checking more than one checkbox is possible*)

- ☐ River
- ☐ Open source wells
- ☐ Bored wells
- ☐ Rain catchment
- ☐ Tap water
- ☐ Treated water
- ☐ Bottled water
- ☐ Other, please specify: ______________________________

21. Do you boil your water before you drink it?

☐ Never ☐ Sometimes ☐ Often ☐ Always

Section VI: Biological factors

22. Do you have contact with soil/sand/floor/pavement/street in your daily activities?

☐ Never ☐ Sometimes ☐ Often ☐ Always

23. When do you have contact with soil/sand/floor/pavement/street?

(*Checking more than one checkbox is possible*)

- ☐ Work related
- ☐ Household related (this includes also contact with floor/pavement during household tasks)
- ☐ Gardening
- ☐ Other, please specify: ______________________________

24. Do you have a cat at home?

☐ Yes (If yes, please answer questions 25, 26, 27, 28, 29 and 30)

☐ No (If no, please continue to question 31)

25. How many cats do you have at home?

☐ 1 ☐ 2 ☐ 3 ☐ 4 ☐ More, please specify: ________

26. Does your cat(s) stay indoor and/or outdoors?

☐ Indoors ☐ Outdoors ☐ Both

27. Do you have a cat litter box for your cat?

☐ Yes ☐ No

28. Do you come in contact/clean the cat litter box/cat faeces?

☐ Every day ☐ Every few days ☐ Weekly ☐ Rarely ☐ Never

29. What do you feed your cat(s)? (*Checking more than one checkbox is possible*)

☐ Commercial dry cat food

☐ Commercial wet cat food

☐ Raw leftovers from the kitchen (including meat)

☐ Well-cooked leftovers from the kitchen (including meat)

☐ It catches its own food

☐ It catches mice/rats

☐ Other, please specify: ____

30. Do you pet your cat?

☐ Never ☐ Sometimes ☐ Often

31. Are there any (stray) cats on your property/in your neighbourhood/work environment?

☐ Never ☐ Sometimes ☐ Often

Section VII: Interview conclusions

This is the end of the interview. Thank you very much for your cooperation

32. Do you have any comments or questions?

____

____

____

____
