# Supplementary material for: Sero-epidemiological status and risk factors of toxoplasmosis in pregnant women in Northern Vietnam
Source: BMC Infect Dis. 2019 Apr 18;19:329. doi: 10.1186/s12879-019-3885-7 (PMC6471880; doi:10.1186/s12879-019-3885-7)
Supplement: Supplementary file 2 — Questionnaire-Vietnamese. (PDF 587 kb) [file 12879_2019_3885_MOESM2_ESM.pdf]

Additional file 2

Tỷ lệ nhiễm huyết thanh và một số yếu tố liên quan đến nhiễm Toxoplasma ở phụ nữ có thai tại một số tỉnh miền Bắc, Việt Nam

CÂU HỎI PHỎNG VẤN

Mã bệnh nhân: [ ]

Người phỏng vấn: ____

Tên bệnh viện: ____

Ngày: ________\\2016

Phần 1: Giới thiệu về nghiên cứu

Mục đích của đề tài này là nghiên cứu về ký sinh trùng *Toxoplasma gondii* ở phụ nữ mang thai. Nghiên cứu nhằm cung cấp các thông tin về tỷ lệ nhiễm, các yếu tố nguy cơ liên quan đến nhiễm bệnh, triệu chứng lâm sàng và cách phòng chống.
Nghiên cứu này đã được Bộ Y tế, Hội đồng Y đức của Viện Y học nhiệt đới Bỉ, Hội đồng y đức đại Học Antwerp Bỉ, Hội đồng Y đức Viện Sốt rét-KST-CT Trung ương phê duyệt.

Phần 2: Thông tin chung của người được phỏng vấn

1. Tuổi: __________________
2. Địa chỉ/mã vùng:...
3. Học vấn:
   - □ Không đi học
   - □ Tiểu học
   - □ Trung học CS
   - □ Trung học phổ thông
   - □ Trung cấp, cao đẳng
   - □ Đại học
   - □ Sau đại học
4. Nghề nghiệp: (*Có thể chọn nhiều câu trả lời*)
   - □ Nội trợ
   - □ Nông dân
   - □ Nghề nghiệp liên quan đến nông nghiệp
   - □ Giúp việc gia đình

- □ Người giết mổ gia súc, gia cầm/hàng xẻ
- □ Nhân viên vệ sinh
- □ Thợ xây, thợ hồ
- □ Nhân viên văn phòng
- □ Kinh doanh

Phần3: Bệnh sử

5. Tuổi thai (*Tính từ ngày đầu kỳ kinh cuối hoặc các biện pháp tính phù hợp)*: _______ tuần
6. Số lần có thai trước đây: ____
7. Số lần bị thai lưu, chết chu sinh: _______________________Vì sao____________________
8. Chị có bị các dấu hiệu này trong trong thời gian mang thai không? (*Có thể chọn nhiều câu trả lời*)
    - □ Triệu chứng của bệnh bạch cầu đơn nhân*(ví dụ: hạch cổ hoặc hạch nách cứng, đối xứng hai bên; triệu chứng giả cúm như sốt, khó chịu, đau cơ, viêm hầu họng, gan lách to)*
    - □ Viêm màng bồ đào *(ví dụ: đau, giảm thị lực, nhìn mờ, mù)*
    - □ Biểu hiện hệ thần kinh trung ương*(Ví dụ: đau đầu, thay đổi trạng thái tâm thần, động kinh, headache, altered mental status, seizures, hôn mê, sốt, mất hoặc giảm chú ý, liệt thần kinh trung ương, bất thường thị giác hoặc động kinh cục bộ)*
    - □ Triệu chứng khác (ghi rõ): ____
    - □ Không có triệu chứng nào như trên.

Phần 4: Nhận thức

9. Chị đã từng nghe về bệnh Toxoplasma chưa? Có ☐ Không ☐
10. Anh chị biết thông tin về bệnh là nhờ đâu? (*Có thể chọn nhiều câu trả lời*)
    - □ Bác sỹ
    - □ Internet
    - □ Bạn bè
    - □ Nguồn khác (ghi rõ): ___________________________

Phần 5: Thói quen ăn uống

11. Chị hay ăn loại thịt gì (mức độ thường xuyên)?
    (*Có thể chọn nhiều câu trả lời*)

- ◻ Thịt lợn ☐ Không bao giờ ☐ Đôi khi ☐ Thường xuyên
- ◻ Thịt bò ☐ Không bao giờ ☐ Đôi khi ☐ Thường xuyên
- ◻ Thịt dê ☐ Không bao giờ ☐ Đôi khi ☐ Thường xuyên
- ◻ Thịt gia cầm ☐ Không bao giờ ☐ Đôi khi ☐ Thường xuyên
- ◻ Cá ☐ Không bao giờ ☐ Đôi khi ☐ Thường xuyên
- ◻ Chó ☐ Không bao giờ ☐ Đôi khi ☐ Thường xuyên
- ◻ Mèo ☐ Không bao giờ ☐ Đôi khi ☐ Thường xuyên
- ☐ Loại khác, ghi rõ: ....☐ Không bao giờ ☐ Đôi khi ☐ Thường xuyên

12. Trung bình mỗi tuần chị ăn thịt mấy lần?

☐ 1 lần☐ 2 lần☐3 lần☐4 lần ☐5 lần☐6 lần☐7 lần☐ Nhiều hơn (ghi rõ): _____

13. Chị chế biến thịt như thế nào?(Có thể chọn nhiều câu trả lời)

- ◻ Tái ☐ Không bao giờ ☐ Đôi khi ☐ Thường xuyên
- ◻ Chín vừa ☐ Không bao giờ ☐ Đôi khi ☐ Thường xuyên
- ◻ Chín kỹ ☐ Không bao giờ ☐ Đôi khi ☐ Thường xuyên
- ◻ Xông khói, lên men, ướp muối ☐ Không bao giờ ☐ Đôi khi ☐ Thường xuyên
- ◻ Nướng ☐ Không bao giờ ☐ Đôi khi ☐ Thường xuyên
- ◻ Chiên ☐ Không bao giờ ☐ Đôi khi ☐ Thường xuyên
- ◻ Cách chế biến khác, ghi rõ: ____

14. Anh chị có bảo quản lạnh thịt trước khi sử dụng không?

☐Không bao giờ ☐ Đôi khi ☐ Thường xuyên ☐ Luôn luôn

15. Chị có ăn rau sống hay sa lát bao giờ không?

☐Không bao giờ ☐ Đôi khi ☐ Thường xuyên ☐Rất thường xuyên

16. Chị có rửa rau quả trước khi ăn sống không?

☐Không bao giờ ☐Đôi khi ☐ Thường xuyên ☐ Luôn luôn

17. Chị thường rửa tay vào những thời điểm nào trong ngày?

(Có thể chọn nhiều câu trả lời)

- ◻ Trước khi ăn
- ◻ Sau khi nấu ăn
- ◻ Sau khi làm vườn
- ◻ Sau khi dọn nhà
- ◻ Sau khi dọn vệ sinh chỗ ở của mèo
- ◻ Sau khi làm việc gây bẩn tay
- ◻ Sau khi đi vệ sinh
- ◻ Không rửa
- ☐ Thời điểm khác, ghi rõ:____

18. Chị dùng gì để rửa tay?

- ◻ Chỉ rửa với nước

- ◻ Nước và xà phòng

19. Chị rửa tay như thế nào?

- ◻ Rửa tay trong chậu nước và nhiều người trong gia đình sử dụng chung một chậu nước
- ◻ Rửa tay dưới vòi nước chảy (trực tiếp từ nước nhà máy)
- ◻ Rửa tay từ bồn chứa có vòi nước chảy
- ◻ Cách khác, ghi rõ: ________________________________

20. Nguồn nước sử dụng trong sinh hoạt hàng ngày của gia đình chị?

(Có thể chọn nhiều câu trả lời)

- ◻ Sông
- ◻ Giếng không có nắp đậy
- ◻ Giếng có nắp đậy
- ◻ Nước mưa
- ◻ Nước vòi
- ◻ Nước đã được sử lý
- ◻ Nước đóng chai
- ◻ Nguồn khác, ghi rõ: ________________________________

21. Chị có đun nước trước khi uống không?

☐Không bao giờ ☐Đôi khi ☐ Thường xuyên ☐ Luôn luôn

Phần 6: Các yếu tố sinh học

22. Chị có thường xuyên phải tiếp xúc với đất trong công việc hàng ngày không?

☐Không bao giờ ☐Đôi khi ☐ Thường xuyên ☐ Luôn luôn

(*Nếu chọn "Không bao giờ" thì chuyển câu 24*)

23. Chị thường phải tiếp xúc với đất vào lúc nào?(có thể chọn nhiều câu trả lời)

- ◻ Khi làm việc (công việc thường xuyên, ví dụ nghề nông)
- ◻ Làm việc nhà
- ◻ Làm vườn
- ◻ Thời điểm khác, ghi rõ: ________________________________

24. Chị có nuôi mèo không?

☐ Có

☐ Không (Chuyển câu 37)

25. Chị nuôi mấy con mèo?

☐1 ☐2 ☐ 3 ☐4 ☐ Khác: ________

26. Mèo nhà chị sống trong hay bên ngoài nhà?

☐ Trong nhà ☐ Bên ngoài ☐ Cả hai

27. Chị có dành chỗ đi vệ sinh riêng (hộp giấy, nhà...) cho mèo không?

☐Có ☐Không

28. Chị có hay vệ sinh hộp giấy, chỗ ở của mèo không?

☐Hàng ngày☐ Vài ngày một lần ☐Hàng tuần ☐Hiếm khi ☐Thỉnh thoảng

29. Thức ăn chị thường sử dụng cho mèo?(có thể chọn nhiều câu trả lời)

- ☐ Thức ăn khô (hộp thức ăn sẵn)
- ☐ Thức ăn ướt (hộp thức ăn sẵn)
- ☐ Thức ăn thừa của gia đình (có cả thịt)
- ☐ Nó tự kiếm ăn (bắt chuột…)
- ☐ Khác, ghi rõ: ____

30. Chị có cho mèo ăn không?

☐Không bao giờ ☐Đôi khi ☐ Thường xuyên

31. Hàng xóm hoặc xung quanh nơi ở, nơi làm việc của chị có hộp hoặc khay vệ sinh của mèo không?

☐Không bao giờ ☐Đôi khi ☐ Thường xuyên

Phần 7: Kết thúc phỏng vấn

Đến đây, các nội dung phỏng vấn đã kết thúc. Xin cảm ơn chị đã nhiệt tình hợp tác.

Chị có ý kiến hay câu hỏi gì thêm không?

____

____

____

____
