# Supplementary material for: Sero-epidemiological status and risk factors of toxoplasmosis in pregnant women in Northern Vietnam
Source: BMC Infect Dis. 2019 Apr 18;19:329. doi: 10.1186/s12879-019-3885-7 (PMC6471880; doi:10.1186/s12879-019-3885-7)
Supplement: Supplementary file 3 — Univariable analysis. (PDF 277 kb) [file 12879_2019_3885_MOESM3_ESM.pdf]

Additional file 3

Univariable analysis of risk factors of toxoplasmosis in pregnant women in Northern Vietnam

The questionnaire was analysed to detect socio-demographic and biologically plausible risk factors associated with toxoplasmosis, awareness, clinical history and presentation of signs and symptoms relating to toxoplasmosis. First the data were assessed for significance using univariable statistics (Additional file 3 Table 1 and 2). A generalized linear model with a binomial distribution and logit link function was applied. For categorical variables, the Pearson's chi-square test was used as a goodness of fit to proof that the observed data did not differ from the theoretical distribution. When any cell value had <5 observations and/or a separation problem occurred for a variable, a logistic regression model using Firth's bias reduction method and the Fisher's exact test was used. Thereafter, variables with a P value under the threshold P≤0.20 were analysed in the multivariable model using a backward selection procedure. The variables that were significant in the univariable and multivariable analysis are summarized in Table 2 and 3 in the manuscript.

Table 1. Univariable analysis of possible demographic and risk factors for toxoplasmosis in Hanoi.

Variable	IgG seronegative Nr (%) or mean (sd)	IgG seropositive Nr (%) or mean (sd)	Odds Ratio (95% CI)	P-value
Demographics:				
Age	27 (5)	28 (5)	1.02 (0.927–1.13)	0.653
Level of education:				0.622[a,b]
College	94 (94%)	6 (6%)	—	
Primary school	1 (100%)	0 (0%)	4.85 (0.049–480)	
Secondary school	59 (98%)	1 (2%)	0.367 (0.059–2.26)	
High school	100 (96%)	4 (4%)	0.651 (0.188–2.25)	
University degree	117 (95%)	6 (5%)	0.804 (0.261–2.48)	
Post university degree	12 (92%)	1 (8%)	1.74 (0.253–12.0)	
Profession:				
Housewife				0.710[a,b]
No	335 (95.2%)	17 (4.8%)	—	
Yes	48 (98.0%)	1 (2.0%)	0.593 (0.107–3.28)	
Farmer				0.386[a,b]
No	301 (95.0%)	16 (5.0%)	—	
Yes	82 (97.6%)	2 (2.4%)	0.554 (0.142–2.16)	
Agricultural sector				1[a,b]
No	369 (95%)	18 (5%)	—	
Yes	14 (100%)	0 (0%)	0.689 (0.036–13.2)	

Slaughter house worker				1[a,b]
No	381 (95%)	18 (5%)	—	
Yes	2 (100%)	0 (0%)	4.12 (0.097–175)	
Butcher				—
No	383 (95.5%)	18 (4.5%)	—	
Street cleaner				0.277[a,b]
No	377 (95.7%)	17 (4.3%)	—	
Yes	6 (85.7%)	1 (14.3%)	4.98 (0.703–35.3)	
Builder				0.242[a,b]
No	378 (95.7%)	17 (4.3%)	—	
Yes	5 (83.3%)	1 (16.7%)	5.90 (0.792–43.9)	
Government employed				0.130[#]
No	229 (97.0%)	7 (3.0%)	—	
Yes	154 (93.3%)	11 (6.7%)	2.34 (0.886–6.16)	
Business woman				1[a,b]
No	337 (95.5%)	16 (4.5%)	—	
Yes	46 (95.8%)	2 (4.2%)	1.10 (0.277–4.36)	
Other				0.382[a,b]
No	352 (95%)	18 (5%)	—	
Yes	31 (100%)	0 (0%)	0.302 (0.017–5.37)	
Clinical history:				
Gestational weeks	12 (1)	13 (1)	2.49 (1.20–5.18)	0.015*[#]
Number of previous pregnancies	1.2 (1.1)	1.3 (1)	1.08 (0.711–1.65)	0.709
Previous stillbirths				0.201[a,b]
No	259 (94.5%)	15 (5.5%)	—	
Yes	124 (97.6%)	3 (2.4%)	0.471 (0.144–1.54)	
Clinical symptoms				1[a,b]
No	358 (95.5%)	17 (4.5%)	—	
Yes	25 (96.2%)	1 (3.8%)	1.21 (0.210–6.90)	
Awareness:				
Heard about toxoplasmosis				1[a,b]
No	375 (95%)	18 (5%)	—	
Yes	8 (100%)	0 (0%)	1.19 (0.056–25.5)	
Meat consumption:				
Pork				1[a,b]
No	1 (100%)	0 (0%)	—	
Yes	382 (96%)	18 (4%)	0.145 (0.002–13.7)	
Beef				1[a,b]
No	20 (100%)	0 (0%)	—	

	Yes	363 (95%)	18 (5%)	2.09 (0.113–38.4)	
Goat					0.602[a,b]
	No	265 (95.0%)	14 (5.0%)	—	
	Yes	118 (96.7%)	4 (3.3%)	0.695 (0.235–2.05)	
Chicken or duck					0.046*[#a,b]
	No	28 (87.5%)	4 (12.5%)	—	
	Yes	355 (96.2%)	14 (3.8%)	0.258 (0.083–0.806)	
Fish					0.206[a,b]
	No	4 (80.0%)	1 (20.0%)	—	
	Yes	379 (95.7%)	17 (4.3%)	0.138 (0.017–1.11)	
Dog					0.852
	No	193 (95.1%)	10 (4.9%)	—	
	Yes	190 (96.0%)	8 (4.0%)	0.813 (0.314–2.10)	
Cat					0.091[#]
	No	324 (96.4%)	12 (3.6%)	—	
	Yes	59 (90.8%)	6 (9.2%)	2.75 (0.992–7.60)	
Frequency meat consumption per week		8 (3)	8 (3)	1.06 (0.906–1.24)	0.471
How meat is consumed:					
Raw					0.671[a,b]
	No	349 (95.6%)	16 (4.4%)	—	
	Yes	34 (94.4%)	2 (5.6%)	1.53 (0.381–6.18)	
Medium rare					0.418
	No	239 (96.4%)	9 (3.6%)	—	
	Yes	144 (94.1%)	9 (5.9%)	1.66 (0.644–4.28)	
Well done					1[a,b]
	No	3 (100%)	0 (0%)	—	
	Yes	380 (95%)	18 (5%)	0.340 (0.011–10.8)	
Cured or smoked or fermented					1[a,b]
	No	334 (95.4%)	16 (4.6%)	—	
	Yes	49 (96.1%)	2 (3.9%)	1.02 (0.259–4.05)	
Bbq					0.620
	No	200 (94.8%)	11 (5.2%)	—	
	Yes	183 (96.3%)	7 (3.7%)	0.695 (0.264–1.83)	
Microwave					0.751
	No	187 (94.9%)	10 (5.1%)	—	
	Yes	196 (96.1%)	8 (3.9%)	0.763 (0.295–1.98)	
Freezing of meat					0.583
	No	75 (93.8%)	5 (6.2%)	—	
	Yes	308 (96.0%)	13 (4.0%)	0.633 (0.219–1.83)	
Fruit and vegetables:					

Raw vegetable fruit or salads consumption				0.652
No	139 (94.6%)	8 (5.4%)	—	
Yes	244 (96.1%)	10 (3.9%)	0.712 (0.275–1.85)	
Washing vegetable fruit or salads before eating raw				1[a,b]
No	20 (100%)	0 (0%)	—	
Yes	363 (95%)	18 (5%)	2.09 (0.113–38.4)	
When hands are washed:				
Before eating				1[a,b]
No	6 (100%)	0 (0%)	—	
Yes	377 (95%)	18 (5%)	0.637 (0.028–14.7)	
Before preparing food				0.613[a,b]
No	21 (100%)	0 (0%)	—	
Yes	362 (95%)	18 (5%)	2.19 (0.120–40.2)	
After gardening				0.914
No	112 (94.9%)	6 (5.1%)	—	
Yes	271 (95.8%)	12 (4.2%)	0.827 (0.303–2.26)	
After cleaning the house or floor or pavement				0.472[a,b]
No	46 (93.9%)	3 (6.1%)	—	
Yes	337 (95.7%)	15 (4.3%)	0.610 (0.182–2.05)	
After cleaning the cat litter box or cat faeces				1
No	275 (95.5%)	13 (4.5%)	—	
Yes	108 (95.6%)	5 (4.4%)	0.979 (0.341–2.81)	
After handling something dirty				0.413[b]
No	38 (92.7%)	3 (7.3%)	—	
Yes	345 (95.8%)	15 (4.2%)	0.493 (0.146–1.67)	
After using the toilet				—
Yes	383 (95.5%)	18 (4.5%)	—	
How hands are washed:				
Hand washing with water and/or soap				1[a,b]
Soap	304 (95.3%)	15 (4.7%)	—	
Water	79 (96.3%)	3 (3.7%)	0.865 (0.263–2.85)	
Where hands are washed:				
Water in basin where hands are washed				0.613[a,b]
No	360 (95%)	18 (5%)	—	
Yes	23 (100%)	0 (0%)	0.415 (0.023–7.53)	
Water from a tap for pouring on hands				0.339
No	161 (97.0%)	5 (3.0%)	—	

	Yes	222 (94.5%)	13 (5.5%)	1.89 (0.659–5.39)	
Water from a container for pouring on hands					0.613
	No	243 (94.9%)	13 (5.1%)	—	
	Yes	140 (96.6%)	5 (3.4%)	0.668 (0.233–1.91)	
Usual source of water					
River					—
	No	383 (95.5%)	18 (4.5%)		
Open source wells					1[a,b]
	No	376 (95%)	18 (5%)	—	
	Yes	7 (100%)	0 (0%)	1.36 (0.061–30.0)	
Bored wells					1
	No	267 (95.4%)	13 (4.6%)	—	
	Yes	116 (95.9%)	5 (4.1%)	0.885 (0.309–2.54)	
Rain catchment					1[a,b]
	No	368 (95%)	18 (5%)	—	
	Yes	15 (100%)	0 (0%)	0.643 (0.034–12.2)	
Tap water					1
	No	110 (95.7%)	5 (4.3%)	—	
	Yes	273 (95.5%)	13 (4.5%)	1.05 (0.365–3.01)	
Treated water					—
	No	383 (95.5%)	18 (4.5%)	—	
Bottled water					1[a,b]
	No	374 (95%)	18 (5%)	—	
	Yes	9 (100%)	0 (0%)	1.07 (0.051–22.1)	
Contact with soil/sand/floor/ pavement/street in daily activities					0.462
	No	256 (96.2%)	10 (3.8%)	—	
	Yes	127 (94.1%)	8 (5.9%)	1.61 (0.621–4.19)	
Context of contact with soil/sand/floor/pavement /street					
Work related					1[a,b]
	No	364 (95%)	18 (5%)	—	
	Yes	19 (100%)	0 (0%)	0.505 (0.027–9.35)	
Household tasks related					0.0733[#]
	No	311 (96.6%)	11 (3.4%)	—	
	Yes	72 (91.1%)	7 (8.9%)	2.75 (1.03–7.34)	
Gardening					1[a,b]
	No	323 (95.6%)	15 (4.4%)	—	
	Yes	60 (95.2%)	3 (4.8%)	1.21 (0.364–4.01)	

Cats				
Cat at home				1
No	276 (95.5%)	13 (4.5%)	—	
Yes	107 (95.5%)	5 (4.5%)	0.992 (0.345–2.85)	
Number of cats	0.43 (0.92)	0.67 (1.33)	1.22 (0.839–1.77)	0.301
Other (stray) cats on property/ in neighbourhood / in work environment				1
No	266 (95.3%)	13 (4.7%)	—	
Yes	117 (95.9%)	5 (4.1%)	0.874 (0.305–2.51)	
Among cat owners:				
Where the cat stays				0.833[a,b]
Both indoor and outdoor	14 (93.3%)	1 (6.7%)	—	
Indoor	41 (95.3%)	2 (4.7%)	0.582 (0.067–5.07)	
Outdoor	52 (96.3%)	2 (3.7%)	0.460 (0.053–3.98)	
Cat litter box				0.676[a,b]
No	55 (96.5%)	2 (3.5%)	—	
Yes	52 (94.5%)	3 (5.5%)	1.48 (0.276–7.95)	
Contact with cat faeces / cleaning the cat litter box				0.605[a,b]
Every day	16 (94.1%)	1 (5.9%)	—	
Every few days	41 (97.6%)	1 (2.4%)	0.398 (0.037–4.31)	
Weekly	12 (92.3%)	1 (7.7%)	1.32 (0.113–15.4)	
Rarely	38 (95.0%)	2 (5.0%)	0.714 (0.083–6.15)	
Never		No cat owner never came in contact with cat faeces / cleans the cat litter box		
Cat food				
Commercial dry cat food				0.544[a,b]
No	92 (95.8%)	4 (4.2%)	—	
Yes	15 (93.8%)	1 (6.2%)	1.99 (0.277–14.3)	
Commercial wet cat food				0.129[#a,b]
No	105 (96.3%)	4 (3.7%)	—	
Yes	2 (66.7%)	1 (33.3%)	14.1 (1.13–175)	
Raw leftovers				1[a,b]
No	106 (95%)	5 (5%)	—	
Yes	1 (100%)	0 (0%)	6.45 (0.064–647)	
Well-cooked leftovers				1[a,b]
No	17 (94.4%)	1 (5.6%)	—	
Yes	90 (95.7%)	4 (4.3%)	0.580 (0.082–4.12)	
Catches its own food				1[a,b]
No	104 (95%)	5 (5%)	—	

Yes	3 (100%)	0 (0%)	2.71 (0.080–92.5)	
Petting of cat				0.666[a,b]
No	50 (94.3%)	3 (5.7%)	—	
Yes	57 (96.6%)	2 (3.4%)	0.627 (0.117–3.37)	

* Significant (P<0.05); # Under the threshold P≤0.20; [a] Obtained using Firth's bias reduction method; [b] Fisher's exact test
Abbreviations: CI, confidence interval; sd, standard deviation

Table 2. Univariable analysis of possible demographic and risk factors for toxoplasmosis in Thai Binh.

Variable	IgG Seronegative Nr (%) or mean (sd)	IgG Seropositive Nr (%) or mean (sd)	Odds Ratio (95% CI)	P-value
Demographics:				
Age	28 (5)	28 (5)	0.977 (0.896–1.07)	0.597
Level of education:				0.157[#a,b]
College	92 (93%)	7 (7%)	—	
Preschool	1 (100%)	0 (0%)	4.11 (0.042–404)	
Primary school	9 (75%)	3 (25%)	4.54 (1.04–19.9)	
Secondary school	94 (97%)	3 (3%)	0.457 (0.124–1.69)	
High school	85 (93%)	6 (7%)	0.938 (0.313–2.81)	
University degree	87 (96%)	4 (4%)	0.634 (0.189–2.13)	
Post university degree	3 (100%)	0 (0%)	1.76 (0.053–58.5)	
Profession:				
Housewife				1[a,b]
No	297 (94.0%)	19 (6.0%)	—	
Yes	74 (94.9%)	4 (5.1%)	0.922 (0.319–2.66)	
Farmer				0.545[a,b]
No	320 (94.4%)	19 (5.6%)	—	
Yes	51 (92.7%)	4 (7.3%)	1.44 (0.490–4.21)	
Agricultural sector				1[a,b]
No	363 (94%)	23 (6%)	—	
Yes	8 (100%)	0 (0%)	0.910 (0.043–19.3)	
Slaughter house worker				1[a,b]
No	368 (94%)	23 (6%)	—	
Yes	3 (100%)	0 (0%)	2.24 (0.071–70.4)	
Butcher				1[a,b]
No	370 (94%)	23 (6%)	—	
Yes	1 (100%)	0 (0%)	5.26 (0.056–495)	
Street cleaner				0.114[#a,b]
No	370 (94.4%)	22 (5.6%)	—	
Yes	1 (50.0%)	1 (50.0%)	16.5 (0.997–272)	

Builder					—
	No	371 (94.2%)	23 (5.8%)	—	
Government employed					0.751
	No	221 (93.6%)	15 (6.4%)	—	
	Yes	150 (94.9%)	8 (5.1%)	0.786 (0.325–1.90)	
Business woman					0.373[a,b]
	No	351 (94.4%)	21 (5.6%)	—	
	Yes	20 (90.9%)	2 (9.1%)	1.99 (0.486–8.18)	
Other					1[a,b]
	No	306 (94.2%)	19 (5.8%)	—	
	Yes	65 (94.2%)	4 (5.8%)	1.08 (0.372–3.13)	
Clinical history:					
Gestational weeks		10 (3)	10 (2)	0.965 (0.815–1.14)	0.674
Number of previous pregnancies		1.2 (1.1)	1.4 (1.1)	1.11 (0.782–1.58)	0.558
Previous stillbirths					0.309
	No	241 (95.3%)	12 (4.7%)	—	
	Yes	130 (92.2%)	11 (7.8%)	1.70 (0.730–3.96)	
Clinical symptoms					1[a,b]
	No	350 (94.1%)	22 (5.9%)	—	
	Yes	21 (95.5%)	1 (4.5%)	1.09 (0.190–6.23)	
Awareness:					
Heard about toxoplasmosis					1[a,b]
	No	358 (94%)	23 (6%)	—	
	Yes	13 (100%)	0 (0%)	0.565 (0.029–10.9)	
Meat consumption:					
Pork					0.0095*[#a,b]
	No	1 (33.3%)	2 (66.7%)	—	
	Yes	370 (94.6%)	21 (5.4%)	0.035 (0.003–0.375)	
Beef					0.274[a,b]
	No	36 (90.0%)	4 (10.0%)	—	
	Yes	335 (94.6%)	19 (5.4%)	0.471 (0.158–1.40)	
Goat					0.824
	No	274 (93.8%)	18 (6.2%)	—	
	Yes	97 (95.1%)	5 (4.9%)	0.785 (0.284–2.17)	
Chicken or Duck					0.114[#a,b]
	No	1 (50.0%)	1 (50.0%)	—	
	Yes	370 (94.4%)	22 (5.6%)	0.061 (0.004–1.00)	
Fish					0.261[a,b]
	No	4 (80.0%)	1 (20.0%)	—	

	Yes	367 (94.3%)	22 (5.7%)	0.184 (0.023–1.45)	
Dog					0.802
	No	143 (93.5%)	10 (6.5%)	—	
	Yes	228 (94.6%)	13 (5.4%)	0.815 (0.348–1.91)	
Cat					0.957
	No	199 (93.9%)	13 (6.1%)	—	
	Yes	172 (94.5%)	10 (5.5%)	0.890 (0.381–2.08)	
Frequency meat consumption per week		10 (3)	9 (4)	0.906 (0.803–1.02)	0.108[#]
How meat is consumed:					
Raw					0.779[a,b]
	No	308 (93.9%)	20 (6.1%)	—	
	Yes	63 (95.5%)	3 (4.5%)	0.829 (0.257–2.68)	
Medium rare					0.319
	No	101 (91.8%)	9 (8.2%)	—	
	Yes	270 (95.1%)	14 (4.9%)	0.582 (0.244–1.39)	
Well done					0.261[a,b]
	No	4 (80.0%)	1 (20.0%)	—	
	Yes	367 (94.3%)	22 (5.7%)	0.184 (0.023–1.45)	
Cured or smoked or fermented					1[a,b]
	No	305 (94.1%)	19 (5.9%)	—	
	Yes	66 (94.3%)	4 (5.7%)	1.06 (0.365–3.08)	
Bbq					1
	No	169 (94.4%)	10 (5.6%)	—	
	Yes	202 (94.0%)	13 (6.0%)	1.09 (0.465–2.54)	
Microwave					0.585
	No	101 (92.7%)	8 (7.3%)	—	
	Yes	270 (94.7%)	15 (5.3%)	0.701 (0.289–1.70)	
Freezing of meat					0.455[a,b]
	No	33 (91.7%)	3 (8.3%)	—	
	Yes	338 (94.4%)	20 (5.6%)	0.580 (0.174–1.93)	
Fruit and vegetables:					
Raw vegetable fruit or salads consumption					1[a,b]
	No	74 (94.9%)	4 (5.1%)	—	
	Yes	297 (94.0%)	19 (6.0%)	1.09 (0.375–3.14)	
Washing vegetable fruit or salads before eating raw					1[a,b]
	No	8 (100%)	0 (0%)	—	
	Yes	363 (94%)	23 (6%)	1.10 (0.052–23.3)	
When hands are washed:					

Before eating				1[a,b]
No	5 (100%)	0 (0%)	—	
Yes	366 (94%)	23 (6%)	0.705 (0.029–17.3)	
Before preparing food				0.404[a,b]
No	27 (90.0%)	3 (10.0%)	—	
Yes	344 (94.5%)	20 (5.5%)	0.468 (0.139–1.58)	
After gardening				0.274[a,b]
No	36 (90.0%)	4 (10.0%)	—	
Yes	335 (94.6%)	19 (5.4%)	0.471 (0.158–1.40)	
After cleaning the house or floor or pavement				0.328[a,b]
No	18 (90.0%)	2 (10.0%)	—	
Yes	353 (94.4%)	21 (5.6%)	0.450 (0.109–1.87)	
After cleaning the cat litter box or cat faeces				1
No	223 (94.1%)	14 (5.9%)	—	
Yes	148 (94.3%)	9 (5.7%)	0.969 (0.409–2.30)	
After handling something dirty				1[a,b]
No	1 (100%)	0 (0%)	—	
Yes	370 (94%)	23 (6%)	0.190 (0.002–17.9)	
After using the toilet				—
Yes	371 (94.2%)	23 (5.8%)	—	
How hands are washed:				
Hand washing with water and/or soap				1
Soap	289 (94.1%)	18 (5.9%)	—	
Water	82 (94.3%)	5 (5.7%)	0.979 (0.353–2.72)	
Where hands are washed:				
Water in basin where hands are washed				1[a,b]
No	366 (94%)	23 (6%)	—	
Yes	5 (100%)	0 (0%)	1.42 (0.058–34.7)	
Water from a tap for pouring on hands				0.802
No	196 (94.7%)	11 (5.3%)	—	
Yes	175 (93.6%)	12 (6.4%)	1.22 (0.526–2.84)	
Water from a container for pouring on hands				0.92
No	181 (93.8%)	12 (6.2%)	—	
Yes	190 (94.5%)	11 (5.5%)	0.873 (0.376–2.03)	
Usual source of water				
River				0.058[#a,b]
No	371 (94%)	22 (6%)	—	
Yes	0 (0%)	1 (100%)	49.5 (0.525–4 670)	

Open source wells					0.385[a,b]
	No	364 (94.3%)	22 (5.7%)	—	
	Yes	7 (87.5%)	1 (12.5%)	3.24 (0.481–21.8)	
Bored wells					0.843
	No	305 (94.4%)	18 (5.6%)	—	
	Yes	66 (93.0%)	5 (7.0%)	1.28 (0.460–3.58)	
Rain catchment					0.492[a,b]
	No	330 (93.8%)	22 (6.2%)	—	
	Yes	41 (97.6%)	1 (2.4%)	0.531 (0.97–2.91)	
Tap water					0.803
	No	80 (93.0%)	6 (7.0%)	—	
	Yes	291 (94.5%)	17 (5.5%)	0.779 (0.297–2.04)	
Treated water					—
	No	371 (94.2%)	23 (5.8%)	—	
Bottled water					1[a,b]
	No	368 (94%)	23 (6%)	—	
	Yes	3 (100%)	0 (0%)	2.24 (0.071–70.4)	
Contact with soil/sand/floor/ pavement/street in daily activities					0.462
	No	80 (92.0%)	7 (8.0%)	—	
	Yes	291 (94.8%)	16 (5.2%)	0.628 (0.250–1.58)	
Context of contact with soil/sand/floor/pavement /street					
Work related					0.753[a,b]
	No	324 (93.9%)	21 (6.1%)	—	
	Yes	47 (95.9%)	2 (4.1%)	0.794 (0.204–3.09)	
Household tasks related					0.762
	No	141 (93.4%)	10 (6.6%)	—	
	Yes	230 (94.7%)	13 (5.3%)	0.797 (0.340–1.87)	
Gardening					0.591[a,b]
	No	300 (93.8%)	20 (6.2%)	—	
	Yes	71 (95.9%)	3 (4.1%)	0.718 (0.223–2.31)	
Cats					
Cat at home					1
	No	228 (94.2%)	14 (5.8%)	—	
	Yes	143 (94.1%)	9 (5.9%)	1.02 (0.432–2.43)	
Number of cats		0.58 (0.89)	0.48 (0.67)	0.867 (0.508–1.48)	0.600
Other (stray) cats on property/ in neighbourhood / in work environment					0.246

No	237 (92.9%)	18 (7.1%)	—	
Yes	133 (96.4%)	5 (3.6%)	0.495 (0.180–1.36)	
Among cat owners:				
Where the cat stays				0.427[a,b]
Both indoor and outdoor	66 (92%)	6 (8%)	—	
Indoor	26 (100%)	0 (0%)	0.193 (0.010–3.74)	
Outdoor	53 (95%)	3 (5%)	0.669 (0.172–2.61)	
Cat litter box				0.292[a,b]
No	93 (95.9%)	4 (4.1%)	—	
Yes	52 (91.2%)	5 (8.8%)	2.18 (0.593–7.98)	
Contact with cat faeces / cleaning the cat litter box				0.506[a,b]
Every day	20 (91%)	2 (9%)	—	
Every few days	46 (98%)	1 (2%)	0.265 (0.032–2.21)	
Weekly	21 (91%)	2 (9%)	0.953 (0.143–6.35)	
Rarely	56 (93%)	4 (7%)	0.653 (0.125–3.42)	
Never	2 (100%)	0 (0%)	1.64 (0.031–85.5)	
Cat food				
Commercial dry cat food				0.107[#a,b]
No	137 (95.1%)	7 (4.9%)	—	
Yes	8 (80.0%)	2 (20.0%)	5.39 (1.03–28.2)	
Commercial wet cat food				0.169[#a,b]
No	134 (95.0%)	7 (5.0%)	—	
Yes	11 (84.6%)	2 (15.4%)	3.90 (0.787–19.3)	
Raw leftovers				1[a,b]
No	140 (94%)	9 (6%)	—	
Yes	5 (100%)	0 (0%)	1.34 (0.053–34.3)	
Well-cooked leftovers				0.107[#a,b]
No	18 (85.7%)	3 (14.3%)	—	
Yes	127 (95.5%)	6 (4.5%)	0.269 (0.066–1.11)	
Catches its own food				0.719[a,b]
No	96 (93.2%)	7 (6.8%)	—	
Yes	49 (96.1%)	2 (3.9%)	0.650 (0.147–2.87)	
Petting of cat				0.467[a,b]
No	45 (91.8%)	4 (8.2%)	—	
Yes	100 (95.2%)	5 (4.8%)	0.553 (0.150–2.04)	

* Significant (P<0.05); [#] Under the threshold P≤0.20; [a] Obtained using Firth's bias reduction method; [b] Fisher's exact test
Abbreviations: CI, confidence interval; sd, standard deviation
